# Supplementary figures and images for: Specific deletion of Axin1 leads to activation of β-catenin/BMP signaling resulting in fibular hemimelia phenotype in mice
Source: eLife. 2022 Dec 21;11:e80013. doi: 10.7554/eLife.80013 (PMC9815809; doi:10.7554/eLife.80013)

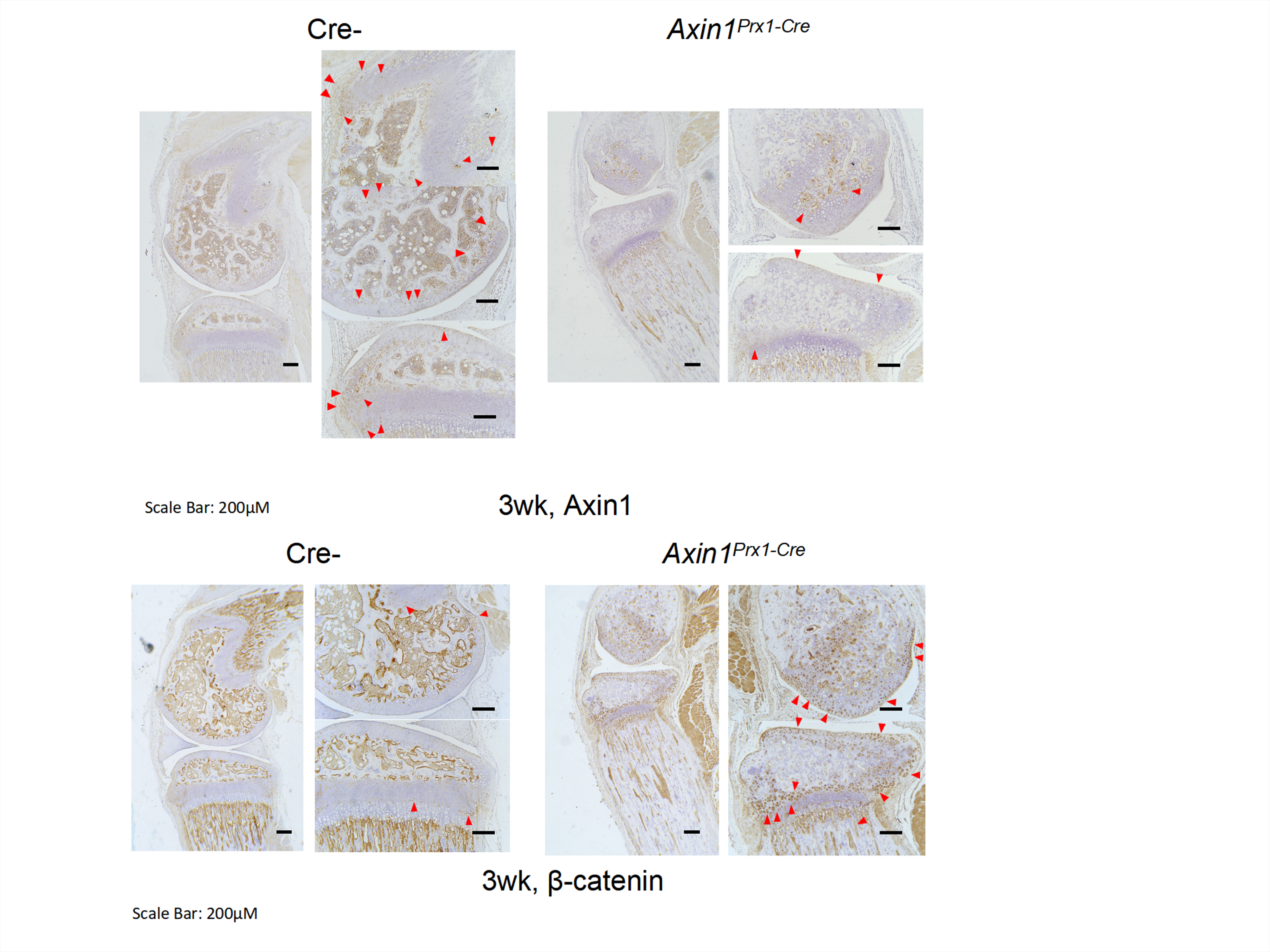

Supplement: Figure 1—source data 1. [file elife-80013-fig1-data1.zip › Figure 1-source data 1 Deletion of Axin1 in limb mesenchymal cells leads to alterations in expression of Axin1 and β-catenin proteins in long bones of.tif]

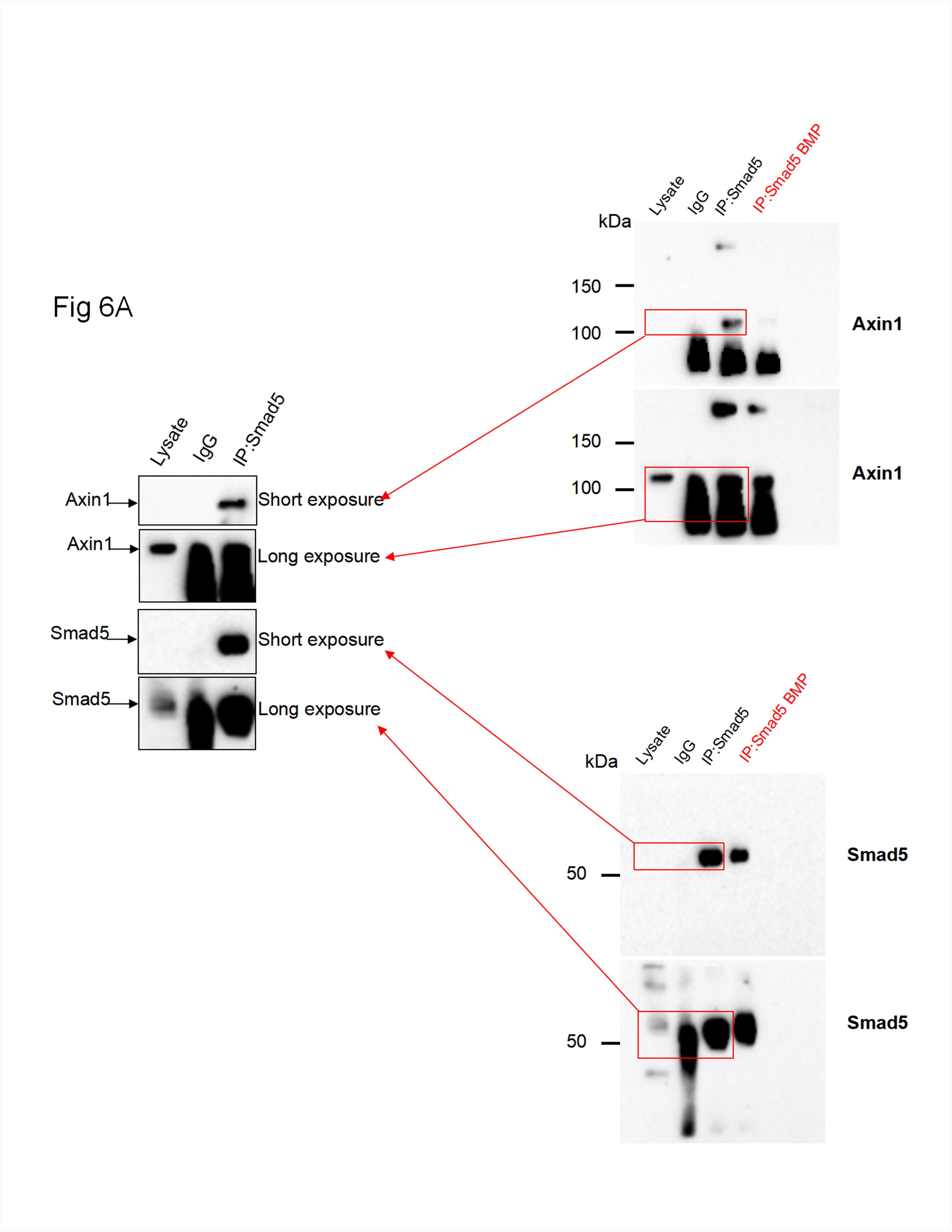

Supplement: Figure 6—source data 1. [file elife-80013-fig6-data1.zip › Figure 6-Source Data 1.tif]

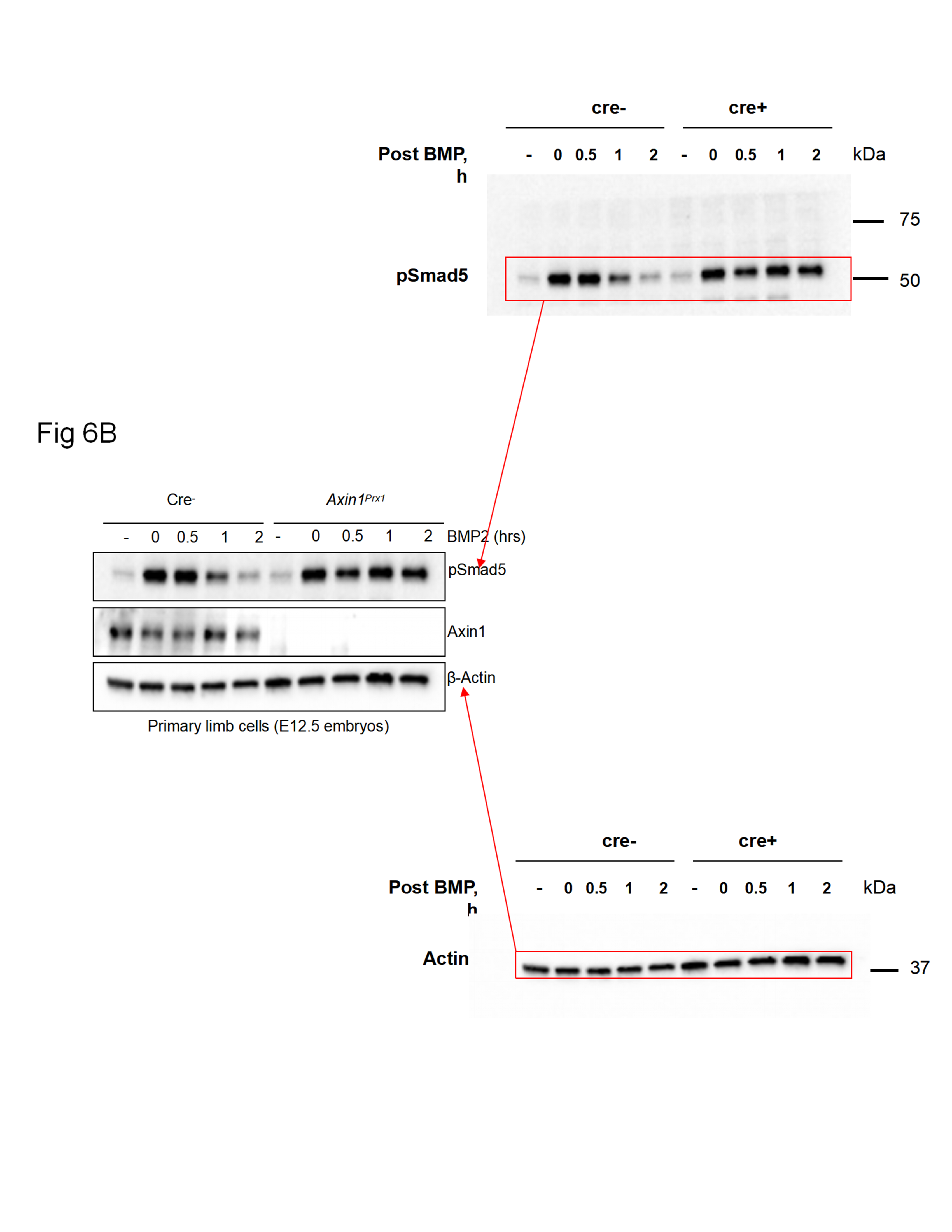

Supplement: Figure 6—source data 2. [file elife-80013-fig6-data2.zip › Figure 6-Source Data 2.tif]

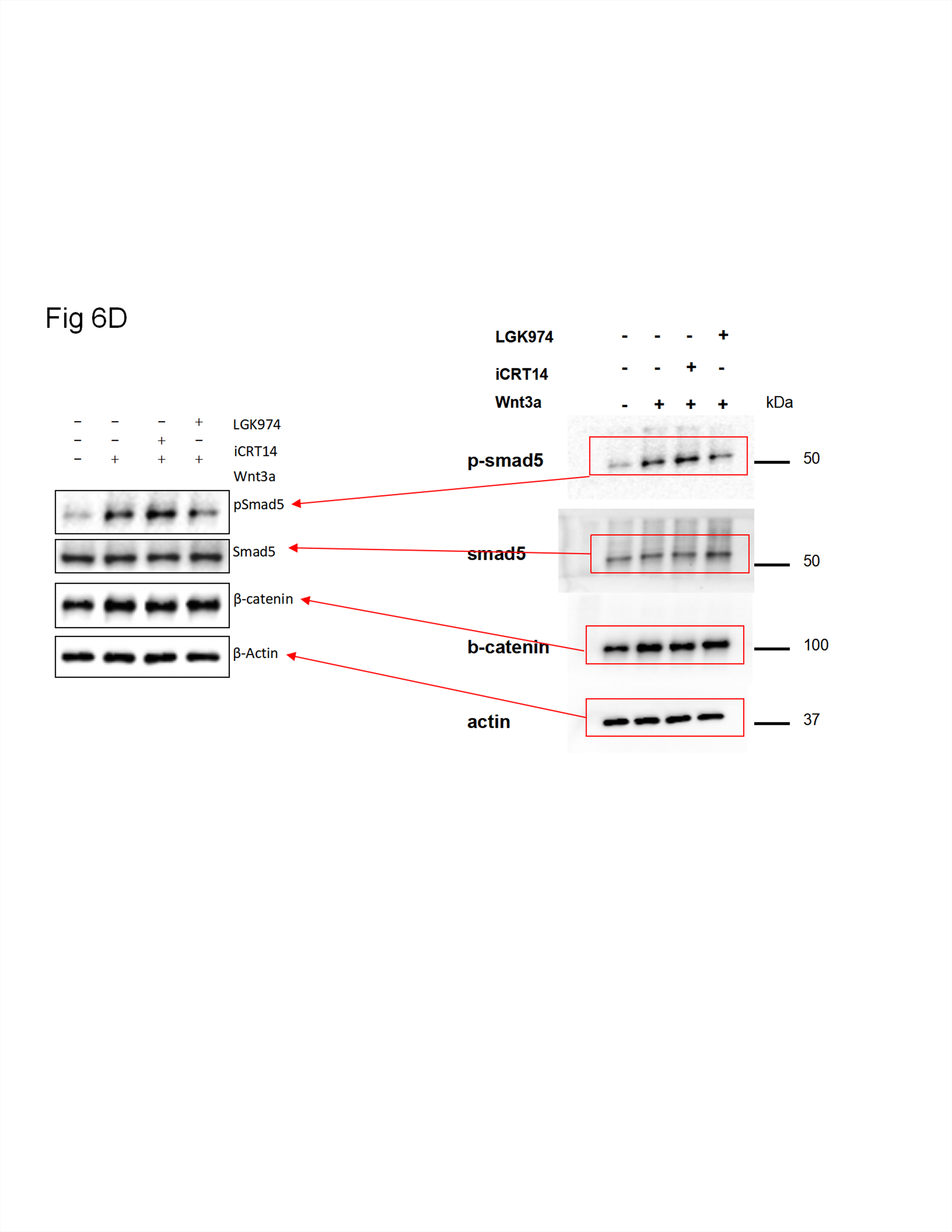

Supplement: Figure 6—source data 4. [file elife-80013-fig6-data4.zip › Figure 6-Source Data 4.tif]

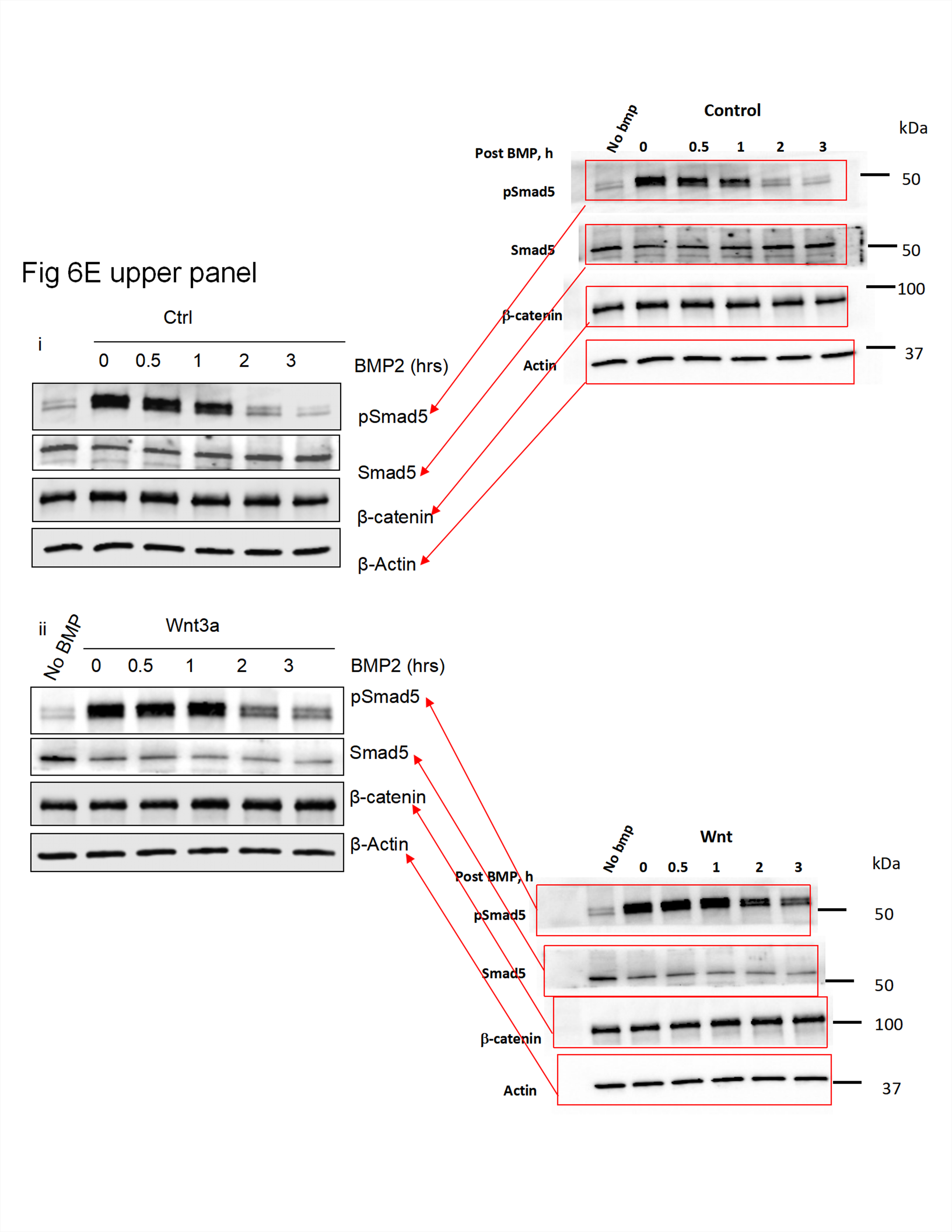

Supplement: Figure 6—source data 5. [file elife-80013-fig6-data5.zip › Figure 6-Source Data 5 upper-1.tif]
