# Supplementary material for: Specific deletion of Axin1 leads to activation of β-catenin/BMP signaling resulting in fibular hemimelia phenotype in mice
Source: eLife. 2022 Dec 21;11:e80013. doi: 10.7554/eLife.80013 (PMC9815809; doi:10.7554/eLife.80013)
Supplement: Figure 2—source data 1. [file elife-80013-fig2-data1.zip › Figure 2-source data 1.pptx]

## Slide 1
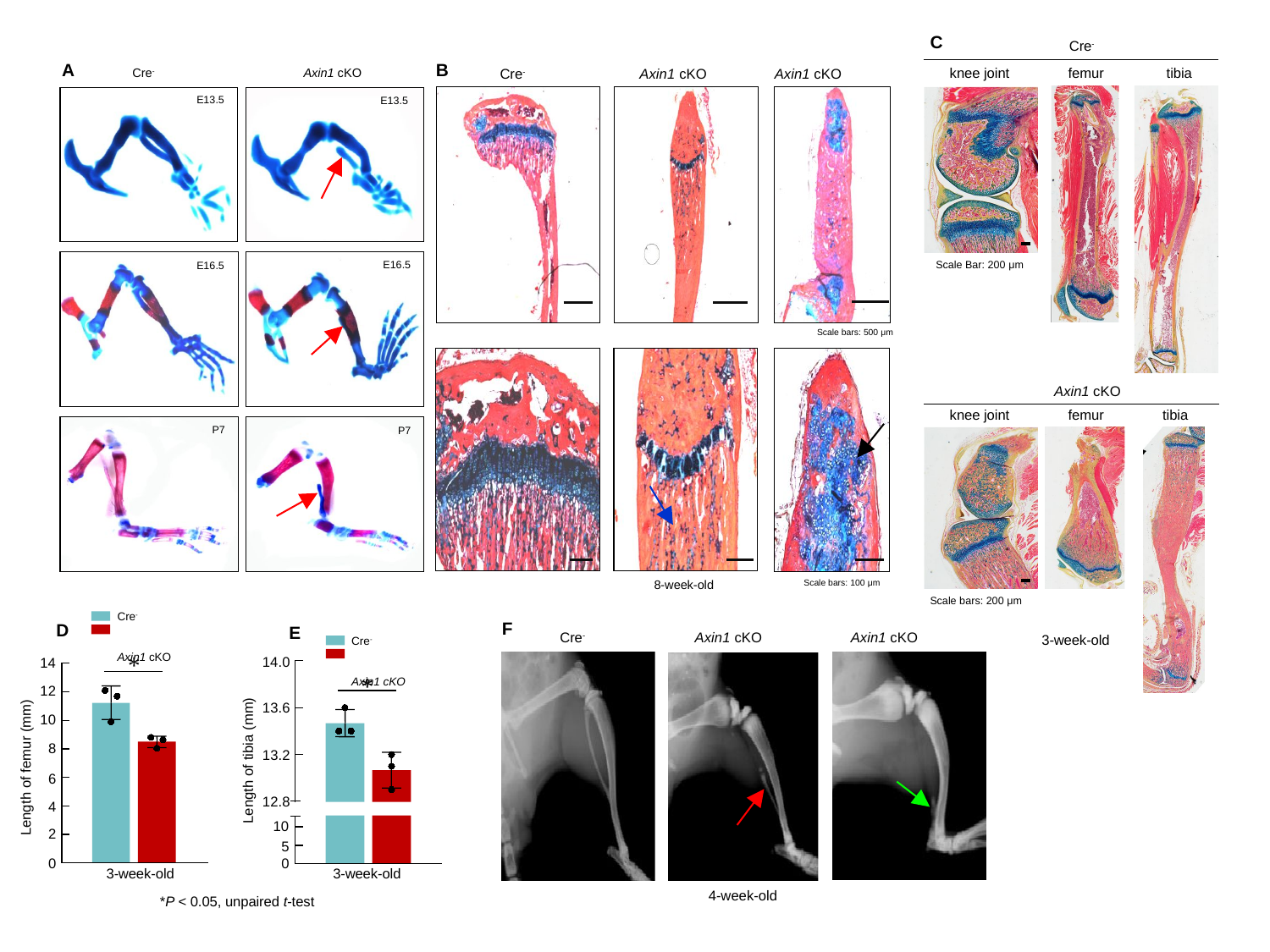

C
Cre-
knee joint femur tibia
Scale Bar: 200 μm
Axin1 cKO
knee joint femur tibia
Scale bars: 200 μm
3-week-old
A
Cre- Axin1 cKO
E13.5
E13.5
E16.5
E16.5
P7
P7
B
Cre- Axin1 cKO Axin1 cKO
Scale bars: 500 μm
8-week-old
Scale bars: 100 μm
Cre- Axin1 cKO
*
14
12
10
 8
Length of femur (mm)
 6
 4
 2
 0
3-week-old
Cre- Axin1 cKO
14.0
*
13.6
13.2
 Length of tibia (mm)
12.8
10
5
0
3-week-old
*P < 0.05, unpaired t-test
F
Cre- Axin1 cKO Axin1 cKO
4-week-old
D
E

## Slide 2
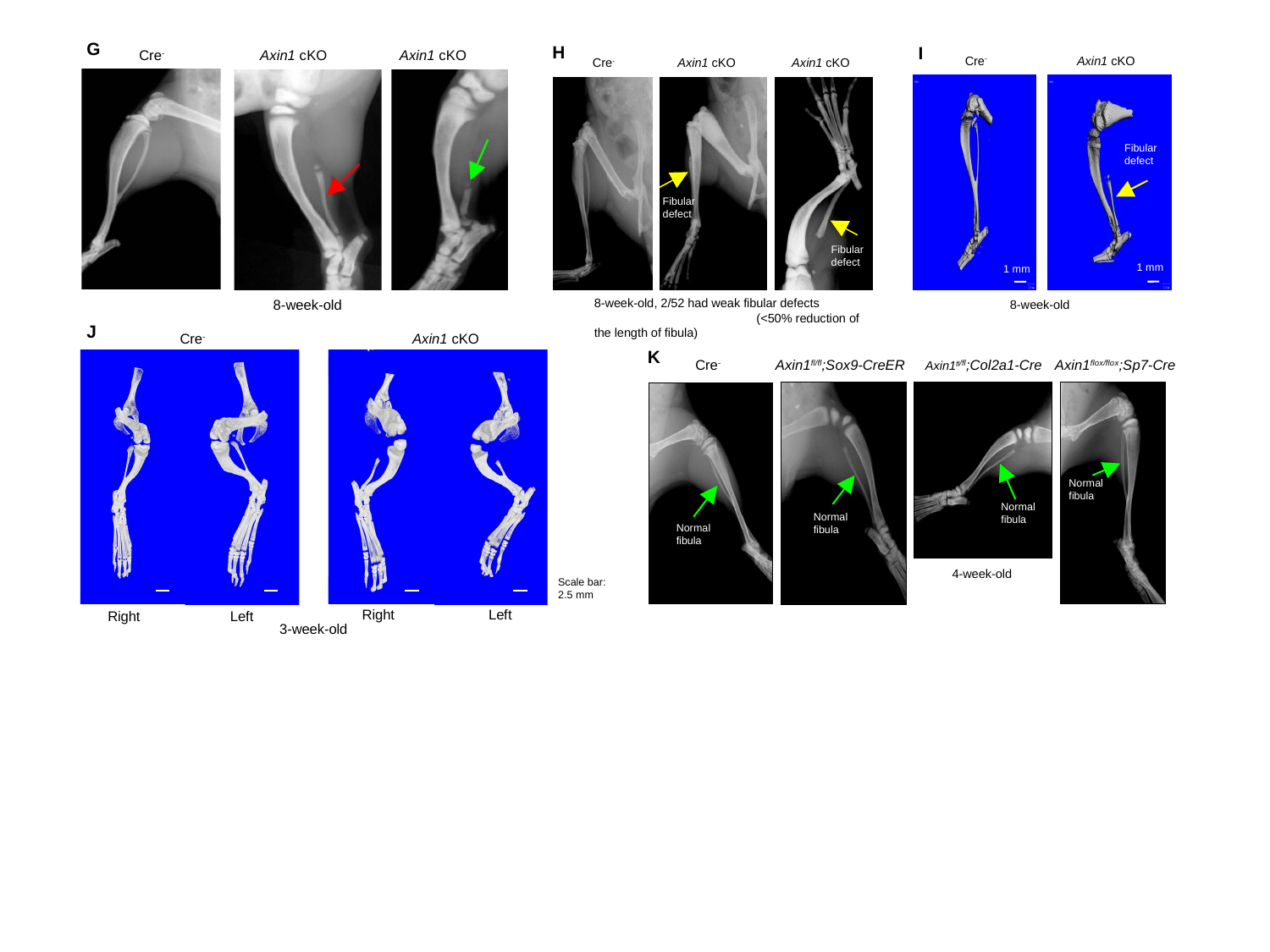

G
Cre- Axin1 cKO Axin1 cKO
8-week-old
H
Cre- Axin1 cKO Axin1 cKO
Fibular defect
Fibular defect
8-week-old, 2/52 had weak fibular defects (<50% reduction of the length of fibula)
I
Cre- Axin1 cKO
Fibular defect
1 mm
1 mm
8-week-old
J
Cre- Axin1 cKO
Scale bar:
2.5 mm
Right Left
Right Left
3-week-old
Cre- Axin1fl/fl;Sox9-CreER Axin1fl/fl;Col2a1-Cre Axin1flox/flox;Sp7-Cre
Normal fibula
Normal fibula
Normal fibula
Normal fibula
4-week-old
K
