# Supplementary material for: Specific deletion of Axin1 leads to activation of β-catenin/BMP signaling resulting in fibular hemimelia phenotype in mice
Source: eLife. 2022 Dec 21;11:e80013. doi: 10.7554/eLife.80013 (PMC9815809; doi:10.7554/eLife.80013)
Supplement: Figure 3—source data 1. [file elife-80013-fig3-data1.zip › Figure 3-source data 1.pptx]

## Slide 1
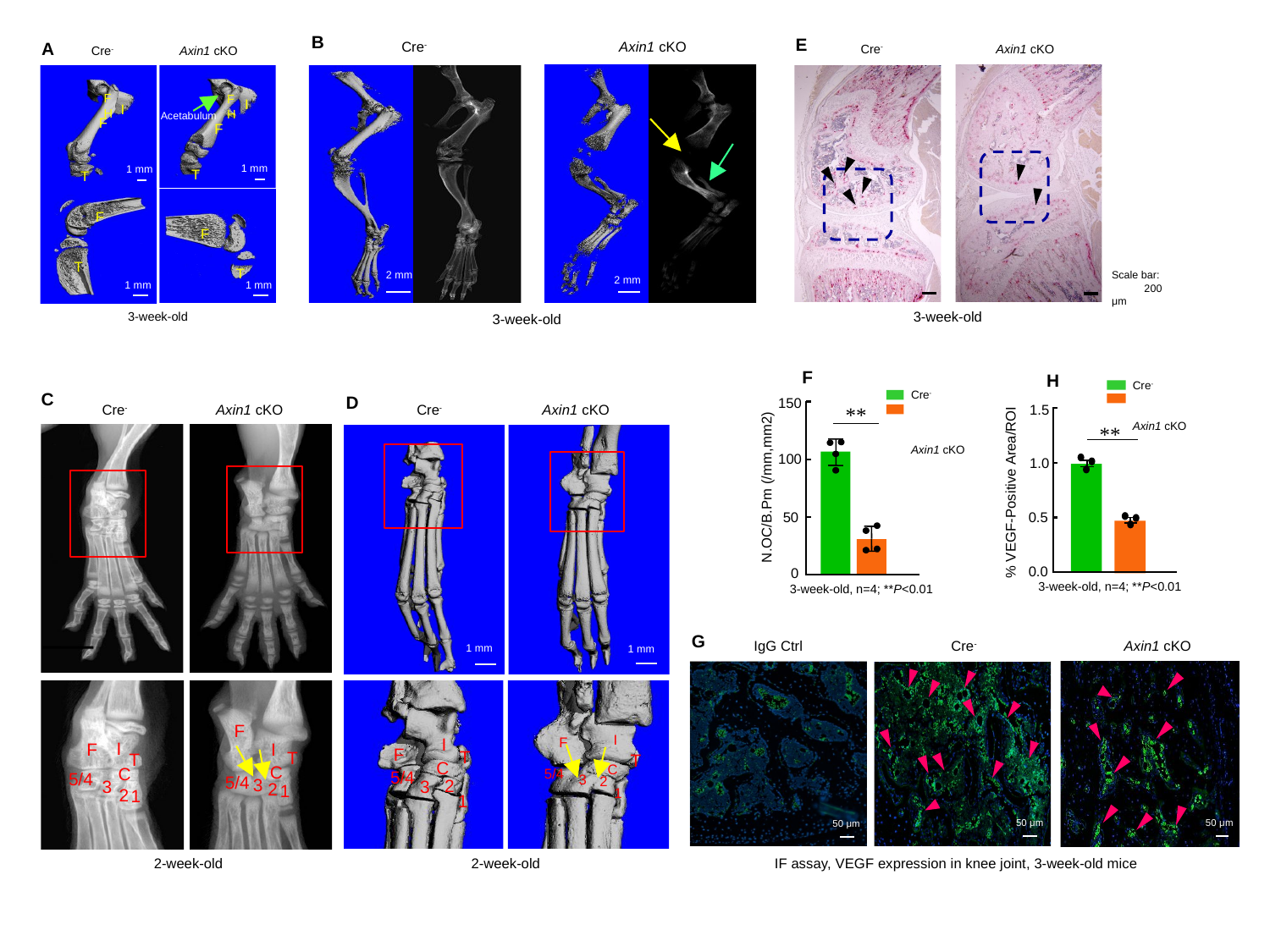

B
Cre- Axin1 cKO
2 mm
2 mm
3-week-old
E
Cre- Axin1 cKO
Scale bar: 200 μm
3-week-old
A
Cre- Axin1 cKO
FH
FH
I
I
Acetabulum
F
F
1 mm
1 mm
T
T
F
F
T
T
1 mm
1 mm
3-week-old
F
Cre- Axin1 cKO
150
**
100
N.OC/B.Pm (/mm,mm2)
50
0
3-week-old, n=4; **P<0.01
H
Cre- Axin1 cKO
1.5
**
1.0
% VEGF-Positive Area/ROI
0.5
0.0
3-week-old, n=4; **P<0.01
C
Cre- Axin1 cKO
F
I
F
T
C
5/4
3
2
1
I
T
C
5/4
3
2
1
2-week-old
D
Cre- Axin1 cKO
1 mm
1 mm
I
I
F
T
C
5/4
2
3
1
F
T
C
5/4
3
2
1
2-week-old
G
IgG Ctrl Cre- Axin1 cKO
50 μm
50 μm
50 μm
IF assay, VEGF expression in knee joint, 3-week-old mice
