# Supplementary material for: Specific deletion of Axin1 leads to activation of β-catenin/BMP signaling resulting in fibular hemimelia phenotype in mice
Source: eLife. 2022 Dec 21;11:e80013. doi: 10.7554/eLife.80013 (PMC9815809; doi:10.7554/eLife.80013)
Supplement: Figure 4—source data 1. [file elife-80013-fig4-data1.zip › Figure 4-source data 1.pptx]

## Slide 1
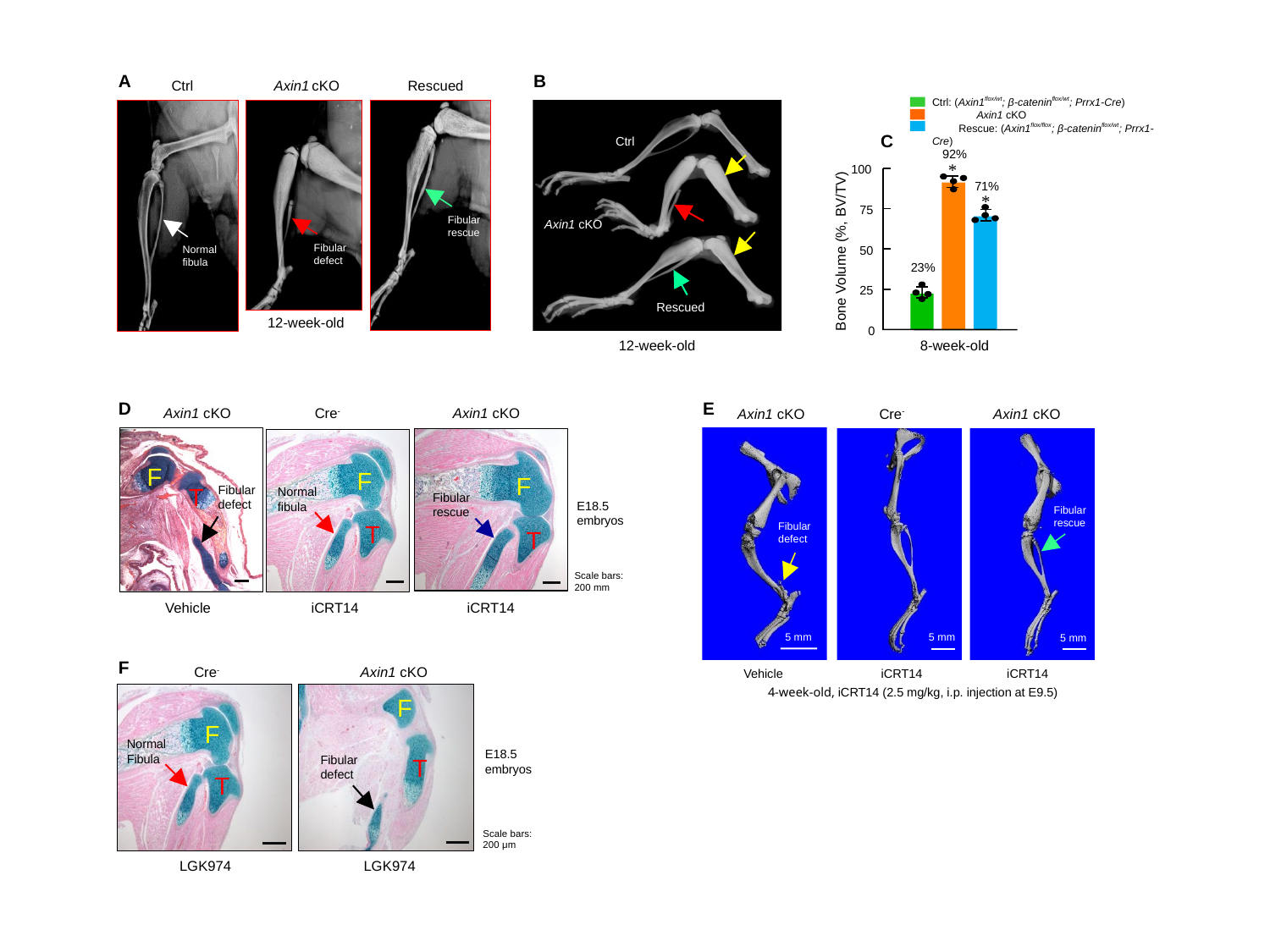

B
Ctrl
Axin1 cKO
Rescued
 12-week-old
A
Ctrl Axin1 cKO Rescued
Fibular defect
Normal fibula
Fibular rescue
12-week-old
Ctrl: (Axin1flox/wt; β-cateninflox/wt; Prrx1-Cre) Axin1 cKO Rescue: (Axin1flox/flox; β-cateninflox/wt; Prrx1-Cre)
C
92%
*
100
71%
*
75
Bone Volume (%, BV/TV)
50
23%
25
0
8-week-old
D
Axin1 cKO Cre- Axin1 cKO
F
F
F
T
Fibular defect
Axin1 KO
Normal fibula
Fibular rescue
E18.5 embryos
T
T
Scale bars: 200 mm
Vehicle iCRT14 iCRT14
E
Axin1 cKO Cre- Axin1 cKO
Fibular rescue
Fibular defect
5 mm
5 mm
5 mm
Vehicle iCRT14 iCRT14
4-week-old, iCRT14 (2.5 mg/kg, i.p. injection at E9.5)
F
Cre- Axin1 cKO
F
F
Normal Fibula
E18.5 embryos
Fibular defect
T
T
Scale bars: 200 μm
LGK974 LGK974
