# Supplementary material for: Specific deletion of Axin1 leads to activation of β-catenin/BMP signaling resulting in fibular hemimelia phenotype in mice
Source: eLife. 2022 Dec 21;11:e80013. doi: 10.7554/eLife.80013 (PMC9815809; doi:10.7554/eLife.80013)
Supplement: Figure 5—source data 6. [file elife-80013-fig5-data6.zip › Figure 5-source data 6.pptx]

## Slide 1
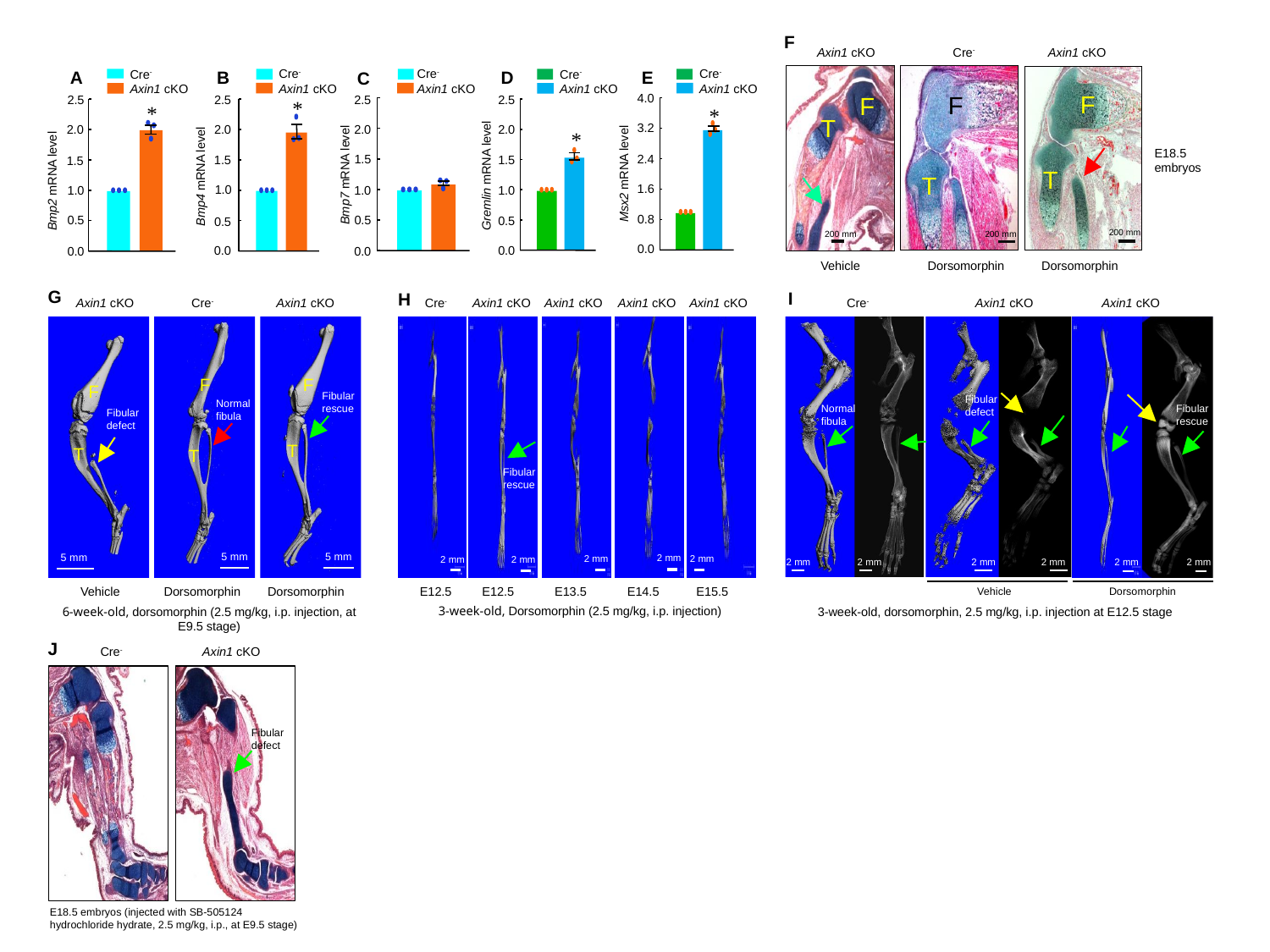

F
Axin1 cKO Cre- Axin1 cKO
F
F
F
T
E18.5 embryos
T
T
200 mm
200 mm
200 mm
Vehicle Dorsomorphin Dorsomorphin
A
Cre- Axin1 cKO
2.5
*
2.0
1.5
Bmp2 mRNA level
1.0
0.5
0.0
Cre- Axin1 cKO
B
*
2.5
2.0
1.5
Bmp4 mRNA level
1.0
0.5
0.0
E
Cre- Axin1 cKO
4.0
*
3.2
2.4
Msx2 mRNA level
1.6
0.8
0.0
Cre- Axin1 cKO
D
2.5
*
2.0
1.5
Gremlin mRNA level
1.0
0.5
0.0
C
2.5
2.0
1.5
Bmp7 mRNA level
1.0
0.5
0.0
G
Axin1 cKO Cre- Axin1 cKO
F
F
F
Fibular rescue
Normal fibula
Fibular defect
T
T
T
5 mm
5 mm
5 mm
Vehicle Dorsomorphin Dorsomorphin
6-week-old, dorsomorphin (2.5 mg/kg, i.p. injection, at E9.5 stage)
I
Cre- Axin1 cKO Axin1 cKO
Fibular defect
Normal fibula
Fibular rescue
2 mm
2 mm
2 mm
2 mm
2 mm
2 mm
 3-week-old, dorsomorphin, 2.5 mg/kg, i.p. injection at E12.5 stage
H
Cre- Axin1 cKO Axin1 cKO Axin1 cKO Axin1 cKO
Fibular rescue
2 mm
2 mm
2 mm
2 mm
2 mm
E12.5 E12.5 E13.5 E14.5 E15.5
3-week-old, Dorsomorphin (2.5 mg/kg, i.p. injection)
Vehicle Dorsomorphin
J
Cre- Axin1 cKO
Fibular defect
E18.5 embryos (injected with SB-505124 hydrochloride hydrate, 2.5 mg/kg, i.p., at E9.5 stage)
Cre- Axin1 cKO
